# A Novel Cross Tetrachiral Honeycomb Metamaterial with Designable Static and Dynamic Performances

**DOI:** 10.3390/ma17184652

**Published:** 2024-09-23

**Authors:** Fengming Liu, Shixuan Shao, Weihan Wang, Rongyu Xia, Mehrdad Negahban, Zheng Li

**Affiliations:** 1 State Key Laboratory for Turbulence and Complex Systems, Department of Mechanics and Engineering Science, College of Engineering, Peking University, Beijing 100871, China; fmliu1678@163.com (F.L.);; 2 Department of Applied Mechanics and Engineering, School of Aeronautics and Astronautics, Sun Yat-sen University, Shenzhen 518107, China; 3 Mechanical and Materials Engineering, University of Nebraska-Lincoln, Lincoln, NE 68588, USA

**Keywords:** cross chiral metamaterial, negative Poisson’s ratio, bandgap, wave isolation, designable properties

## Abstract

A novel cross tetrachiral honeycomb metamaterial is proposed, which not only possesses the negative Poisson’s ratio property, but also has a wide-frequency bandgap. The effective elastic parameters of the cross tetrachiral honeycomb are first theoretically analyzed; then, its designable performances for negative Poisson’s ratio and elastic modulus are studied by varying geometric parameters. The dynamic properties of the cross tetrachiral honeycomb metamaterial are investigated by analyzing the band structure. It is shown that without the addition of external mass to the structure, a designable wide bandgap can be generated to isolate the in-plane waves effectively by selecting the ligament angles and the radius of central cylinder. In addition, an effective approach is proposed for tuning the bandwidth without changing the geometric parameters of the structure. Compared to classical negative Poisson’s ratio metamaterials, the proposed cross tetrachiral honeycomb metamaterial is designable and tunable for achieving a specific static or dynamic performance, and has potential applications in engineering practice.

## 1. Introduction

Mechanical metamaterials are artificially designed materials with mesoscale or microscale structural arrangements, and have extraordinary properties that are not found in natural materials. Their mechanical properties do not just depend on their material properties, but can be designed by the mesoscale or microscale structures [1,2,3]. Therefore, metamaterials have a variety of structural types obtained through designs to meet broad engineering requirements in defense and military, aerospace, civil construction, vehicle manufacturing, shipbuilding engineering, and other industries. Metamaterials have been used in some practical applications in specific projects such as the skin filling of deformable airfoils [4], automotive comfort optimization [5], clinical diagnostics, and biomedical testing [6].

Based on elaborate structural designs, mechanical metamaterials are adjustable [7] and programmable [8,9,10], and can achieve unique physical and mechanical properties [11,12,13,14], such as negative compressibility [15], negative thermal expansion [16], negative Poisson’s ratio [17], and mechanical vibration isolation [18]. Among these, metamaterials with negative Poisson’s ratio, i.e., auxetic materials [19], have attracted more attention, and many structures with this property have been reported, including trichiral and anti-trichiral, tetrachiral and anti-tetrachiral, and hexachiral honeycomb structures [20,21,22]. Beyond the negative Poisson’s ratio, the dynamic behaviors of these metamaterials have also been explored. They have shown good performances in impact resistance [23], fracture tolerance [24], energy absorption, and vibration isolation [25].

Yajun Xin et al. [26] chose a star-shaped negative Poisson’s ratio structure, and proposed a novel re-entrant method to adjust low frequency bandgaps by adding rubber-coated masses. As a typical type of auxetic metamaterial, the chiral honeycomb metamaterial has been developed to obtain both excellent mechanical properties and vibration isolation. Luyun Chen et al. [27] have analyzed the geometric parameters and material properties of hexachiral metamaterials to obtain a wide and complete bandgap. However, most of the studies in this area focus on the generation of bandgaps by additional mass. Xiaoning Liu et al. [28] added a softly coated heavy cylinder into the two-dimensional periodic chiral lattice to achieve low-frequency bandgaps. Pei Sun et al. [29] proposed an internal resonance unit with cruciate ligament features to replace the metamaterial inclusion for extreme low-frequency bandgaps. Similarly, based on a chiral lattice, Rui Zhu et al. [18] first designed an elastic metamaterial beam with distributed multiple inner resonators for achieving wide-band vibration suppression without sacrificing the load-bearing capacity of the beam. Furthermore, four-ligament chiral honeycomb structures have attracted more attention for their better specific energy absorption than other honeycomb structures [30]. Andrea Bacigalupo et al. [31] numerically optimized the designs of metamaterial beams with tetrachiral, tetrachiral backhander, and hexachiral chiral honeycombs to obtain the best dispersion spectra, and used adaptive surrogate-based optimization technology to design a double quad-ligamentous chiral metamaterial with broadband low-frequency filtering [32]. Although beyond negative Poisson’s ratio, the chiral honeycomb metamaterials can effectively reduce vibration at locally resonant bandgaps, most designs use composite materials for complex configurations, resulting in localized resonance by adding extra mass, which leads to higher manufacturing costs, narrower bandgap width, and difficulty in flexible adjustment. As such, a design using a single material and integrated configuration is of significance to engineering applications. In addition, the metamaterial design methodology to satisfy both static and dynamic performance criterion is significant.

In this paper, inspired by the tetrachiral honeycomb and cross chiral structures, a novel cross tetrachiral honeycomb metamaterial with negative Poisson’s ratio is presented that is capable of wave isolation. Different from the usual approach of metamaterials to form a localized resonant bandgap by introducing additional mass, the cross tetrachiral honycomb metamaterial forms a wide bandgap by introducing more geometrical parameters including tile angle *θ*. The metamaterial can be geometrically and mechanically tuned to adjust the broadband bandgap while ensuring excellent static properties. By introducing more geometric parameters, the metamaterial can adjust the broadband bandgap through geometric regulation and mechanical regulation, while ensuring an excellent static performance. The remainder of the paper is arranged as follows. In Section 2, the effective elastic parameters of the proposed metamaterials are theoretically analyzed. In Section 3, three dimensionless quantities are introduced to describe the designability of static machinal response. In Section 4, the dynamic performance of the proposed metamaterial is studied through band structure analysis. Bandgaps have been designed through the selection of geometric parameters and can be tuned by external loading. In Section 5, conclusions are provided to summarize the design and analysis of cross tetrachiral honeycomb metamaterials.

## 2. Cross Tetrachiral Honeycomb Metamaterials

### 2.1. Model

**Figure 1:**
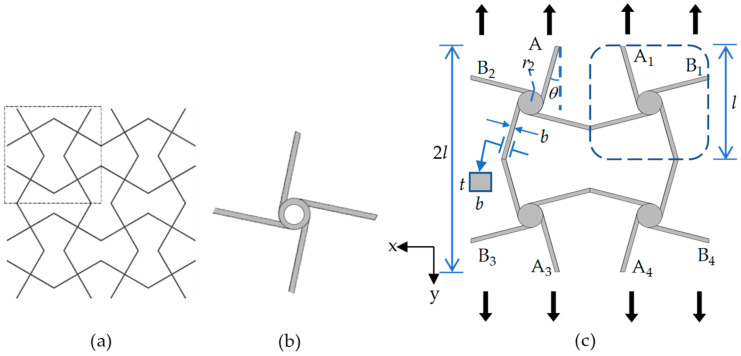
(**a**) The cross chiral honeycomb structure; (**b**) tetrachiral honeycomb; (**c**) unit cell of cross tetrachiral honeycomb metamaterial.

In order to improve the deformability of tetrachiral honeycomb structures, a cross-chiral honeycomb structure [33,34,35] with folded corners, as shown in Figure 1a, is introduced into the classical tetrachiral honeycomb structure with the unit cell shown in Figure 1b. For classical traditional tetrachiral honeycomb structures, there is a circular ring at the center of the ligaments to achieve a light weight structure, but it also reduces the stiffness of the unit cell. This weakness affects the negative Poisson’s ratio and the structural stability in large deformations. To overcome this drawback, we replace the circular ring with a solid cylinder, and attach four identical ligaments tangentially to the cylinder to form an elementary unit, as marked by the blue dashed rectangular box in Figure 1c. By translating and mirroring the elementary unit, a unit cell of cross tetrachiral honeycomb metamaterial is formed, as shown in Figure 1c. The geometrical configuration of the unit cell can be described by the length of the elementary unit *l*, the tilt angle of the ligament *θ*, the radius of the solid cylinder *r*, the in-plane thickness *t*, and the cross-section width *b* of the ligaments, as shown in Figure 1c.

### 2.2. Effective Elastic Parameters

The symmetry of the structure formed from the unit cell (Figure 1c) indicates that the mechanical properties of the unit cell along the *x* and *y* directions are identical. Therefore, only the elastic properties in one direction (either *x* or *y*) need to be analyzed to describe the structure’s behavior.

**Figure 2:**
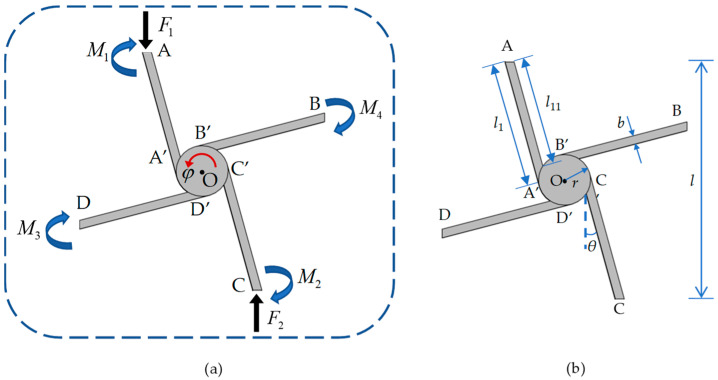
(**a**) Force diagram of elementary unit; (**b**) geometric parameters elementary unit.

Here, we consider small deformations consistent with linear elasticity, a rigid solid cylinder, and the ligaments analyzed using Euler–Bernoulli beam theory, as well as assuming that the deformations are in plane. It is worth noting that when the metamaterial undergoes an overall large deformation, it is actually a result of the cumulative effect of the bending deformations of the ligaments and the rotations of the cylinders. The strain generated locally in each ligament remains as a small deformation that satisfies the Euler–Bernoulli theory. Based on the geometric symmetry, when the unit cell is subject to a uniformly distributed load in the *x* or *y* directions obtained by an action on boundary points A_1_~A_4_ or B_1_~B_4_, respectively, only surface translations are observed. Suppose the unit cell is loaded in the *y* direction and eliminates the rigid body displacement of the center of the unit cell. In order to simplify the analysis, only one elementary unit in the unit cell in Figure 1c is considered, as shown in Figure 2.

The loading of the elementary unit is shown in Figure 2a, where *F*_1_ and *F*_2_ are externally applied loads along the *y* direction, respectively, on endpoints A and C; *M*_1_, *M*_2_, *M*_3_, and *M*_4_ are moments applied, respectively, on endpoints A, B, C, and D. Because the ligaments are tangentially attached to the cylinder, its lengths of two sides are not equal and can be separately named *l*_11_ and *l*_1_, as shown in Figure 2b.

Using the center point O of the solid cylinder as the fixed reference point with the assumed displacements $u_{O}=v_{O}=0$ where *u* denotes displacement in the *x* direction and *v* denotes the displacement in the *y* direction, under the action of the external loads, the solid cylinder will have a rigid body rotation with a rotation angle *φ* around point O.

According to the force equilibrium along the *y* direction, we obtain $F_{1}=F_{2}$, and through the symmetry, the moment has the relations of $M_{1}=M_{2}$ and $M_{3}=M_{4}$. And then, the moment equilibrium equations in plane can be derived using the following equation:

(1) $$F_{1}\left(l_{11}\sin\theta +r\cos\theta \right)=M_{1}+M_{4}.$$

**Figure 3:**
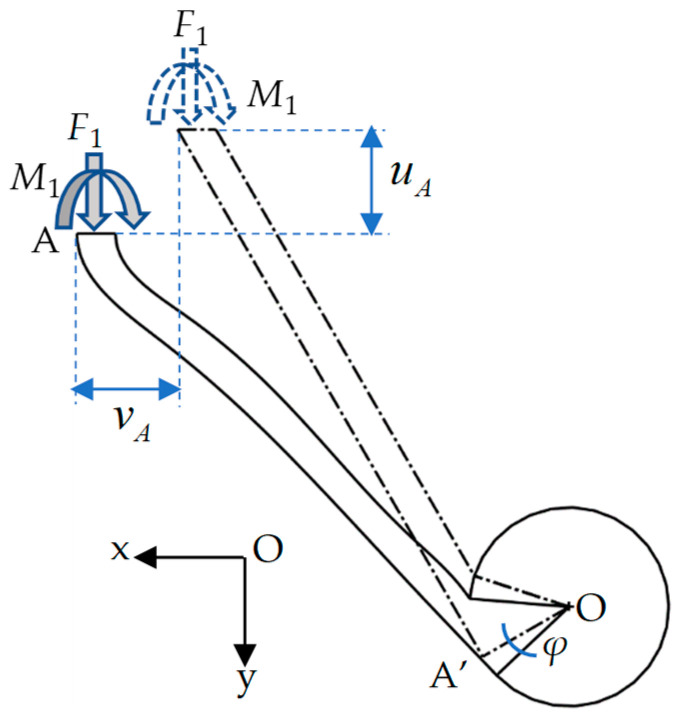
Deformations of ligament AA’ under actions of external load *F*_1_ and moment *M*_1_.

It is clear that the deformations of ligaments AA’ and CC’ are the same due to the symmetry, and so are the ligaments BB’ and DD’. Therefore, the deformation of the elementary unit can be divided into two parts for analysis. The deformation of ligament AA’, as shown in Figure 3, is first taken into consideration.

**Figure 4:**
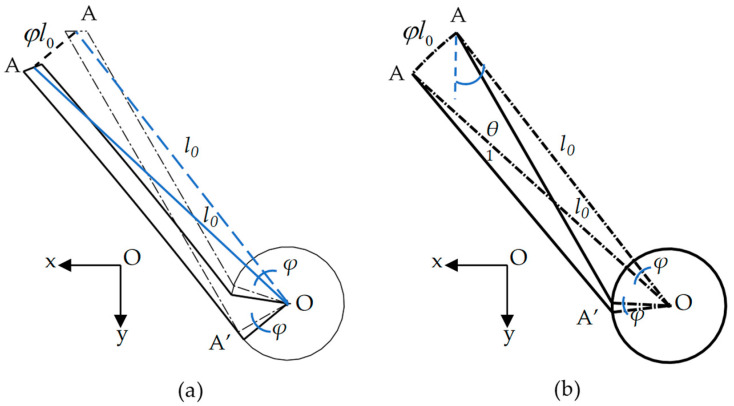
Displacement of ligament AA’ induced by cylinder rotation: (**a**) schematic diagram; (**b**) geometric relations between deformed and undeformed ligaments.

Under the actions of force *F*_1_ and moment *M*_1_, the displacements of ligament AA’ include axial tension/compression and bending deformations, as well as the rotation *φ* of the rigid cylinder. According to Figure 2b, the lengths of ligament AA’, named *l*_11_ and *l*_1_, have the following relations:

(2) $$l_{1}=\frac{2r\sin\theta +l}{2\cos\theta }=\left(r_{q}\tan\theta +\frac{1}{2\cos\theta }\right)l=\alpha l,$$

and

(3) $$l_{11}=l_{1}-r\sin\left(\arccos\left(1-\frac{b}{r}\right)\right)-b\tan\theta =\left(\alpha -r_{q}\sin\left(\arccos\left(1-\frac{b_{q}}{r_{q}}\right)\right)-b_{q}\tan\theta \right)l=\beta l,$$

where $\alpha (r_{q},\theta )$ and $\beta \left(r_{q},b_{q},\theta \right)$ can be represented by the dimensionless parameters $r_{q}=\frac{r}{l}$ and $b_{q}=\frac{b}{l}$ with the length of elementary unit *l*. If we set $l_{11}$ to be the equivalent length of the ligament, the displacements at point A can be expressed as follows:

(4) $$u_{A}=\frac{F_{1}{l_{11}}^{3}\sin\theta \cos\theta }{3EI}-\frac{M_{1}{l_{11}}^{2}\cos\theta }{2EI}-\frac{F_{1}l_{11}\sin\theta \cos\theta }{EA}+\varphi l_{0}\cos\theta_{1},$$

and

(5) $$v_{A}=\frac{F_{1}{l_{11}}^{3}\sin^{2}\theta }{3EI}-\frac{M_{1}{l_{11}}^{2}\sin\theta }{2EI}+\frac{F_{1}l_{11}\cos^{2}\theta }{EA}+\varphi l_{0}\sin\theta_{1},$$

where *E* is the elastic modulus, $I=tb^{3}/12$ is the moment of inertia of the ligament, A=tb is the area of the cross-section of the ligament, and $l_{0}$ is the length from the center O of the cylinder to the endpoint A to be $l_{0}=\sqrt{{l_{1}}^{2}+r^{2}}$. As shown in Figure 4a, the rotation of the cylinder can cause the displacement of the ligament at the endpoint A to be $\varphi l_{0}$. If we introduce a new tilt angle $\theta_{1}$ between OA and ligament AA’, as shown in Figure 4b, its relation with *θ* is $\theta_{1}=\theta +\arctan\frac{r}{l_{1}}=\theta +\arctan\frac{r_{q}}{\alpha }$.

The rotation of endpoint A should be equal to 0 due to the symmetric conditions, i.e.,

(6) $$\frac{F_{1}{l_{11}}^{2}\sin\theta }{2EI}-\frac{M_{1}l_{11}}{EI}+\varphi =0.$$

Then, the displacements at point A can be expressed in terms of external load $F_{1}$, as follows:

(7) $$u_{A}=F_{1}\left(\frac{l_{11}r\cos\theta }{2EI}\left(l_{0}\cos\theta_{1}-\frac{l_{11}\cos\theta }{2}\right)+\frac{{l_{11}}^{2}\sin\theta }{4EI}\left(l_{0}\cos\theta_{1}-\frac{l_{11}\cos\theta }{6}\right)-\frac{l_{11}\sin\theta \cos\theta }{EA}\right),$$

and

(8) $$v_{A}=F_{1}\left(\frac{l_{11}r\cos\theta }{2EI}\left(l_{0}\sin\theta_{1}-\frac{l_{11}\sin\theta }{2}\right)+\frac{{l_{11}}^{2}\sin\theta }{4EI}\left(l_{0}\sin\theta_{1}-\frac{l_{11}\sin\theta }{6}\right)+\frac{l_{11}\cos^{2}\theta }{EA}\right).$$

**Figure 5:**
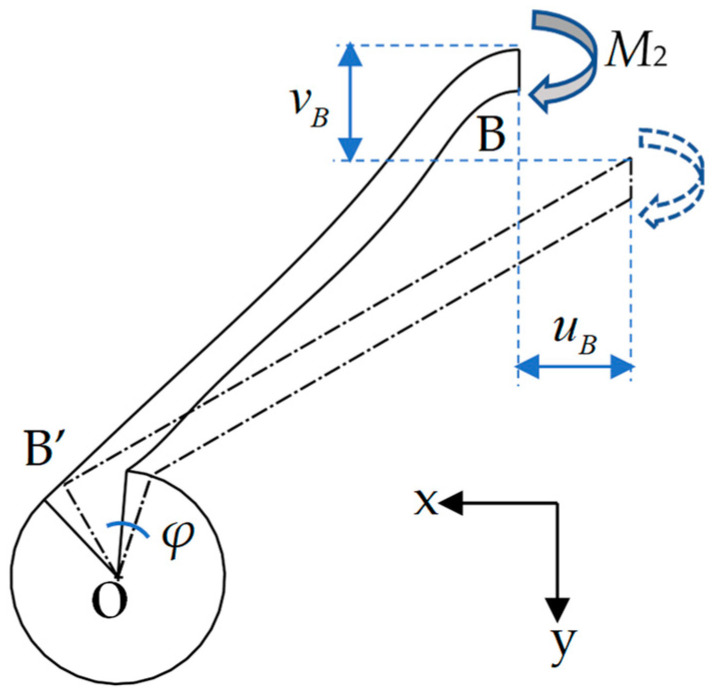
Deformation of ligament BB’ under the action of moment *M*_4_.

Similarly, for the deformation of ligament BB’, as illustrated in Figure 5, the displacements of endpoint B can be obtained using the following equations:

(9) $$u_{B}=\frac{M_{4}{l_{11}}^{2}\sin\theta }{2EI}-\varphi l_{0}\sin\theta_{1},$$

(10) $$v_{B}=-\frac{M_{4}{l_{11}}^{2}\cos\theta }{2EI}+\varphi l_{0}\cos\theta_{1}.$$

Considering Equations (1) and (6) and the symmetry conditions at the endpoint B with $\frac{M_{2}l_{11}}{EI}-\varphi =0$, the displacements of endpoint B can be expressed by the external load $F_{1}$ as an independent variable, i.e.,

(11) $$u_{B}=-F_{1}\frac{l_{11}}{2EI}\left(r\cos\theta +\frac{l_{11}\sin\theta }{2}\right)\left(l_{0}\sin\theta_{1}-\frac{l_{11}\sin\theta }{2}\right),$$

(12) $$v_{B}=F_{1}\frac{l_{1}}{2EI}\left(r\cos\theta +\frac{l_{1}\sin\theta }{2}\right)\left(l_{0}\cos\theta_{1}-\frac{l_{1}\cos\theta }{2}\right).$$

Thus, combining the displacements of endpoints A and B in Equations (9)–(12), for the elementary unit, the equivalent Poisson’s ratio and elastic modulus can be obtained using the following equations:

(13) $$\nu_{q}=\frac{2u_{B}}{l}/\left(\frac{2v_{A}}{l}\right),$$

and

(14) $$E_{s}=\frac{2F_{1}}{lt}/\left(\frac{4v_{A}}{l}\right).$$

Furthermore, we can define the equivalent Poisson’s ratio and elastic modulus in a dimensionless expression as follows:

(15) $$\begin{array}{l} \nu_{q}\left(r_{q},b_{q},\theta \right)=\\ \frac{-\left(r_{q}\cos\theta +\frac{\beta \sin\theta }{2}\right)\left(\sqrt{\alpha^{2}+{r_{q}}^{2}}\sin\theta_{1}-\frac{\beta \sin\theta }{2}\right)}{r_{q}\cos\theta \left(\sqrt{\alpha^{2}+{r_{q}}^{2}}\sin\theta_{1}-\frac{\beta \sin\theta }{2}\right)+\frac{\beta \sin\theta }{2}\left(\sqrt{\alpha^{2}+{r_{q}}^{2}}\sin\theta_{1}-\frac{\beta \sin\theta }{6}\right)+\frac{{b_{q}}^{2}\cos^{2}\theta }{6}}, \end{array}$$

and

(16) $$\begin{array}{l} E_{q}\left(r_{q},b_{q},\theta \right)=\frac{E_{s}}{E}\\ =\frac{{b_{q}}^{3}}{2\beta \left(6r_{q}\cos\theta \left(\sqrt{\alpha^{2}+{r_{q}}^{2}}\sin\theta_{1}-\frac{\beta \sin\theta }{2}\right)+3\beta \sin\theta \left(\sqrt{\alpha^{2}+{r_{q}}^{2}}\sin\theta_{1}-\frac{\beta \sin\theta }{6}\right)+{b_{q}}^{2}\cos^{2}\theta \right)}. \end{array}$$

Therefore, elastic parameters, i.e., equivalent Poisson’s ratio and elastic modulus, can be determined by three geometric dimensionless quantities—*b_q_*, *r_q_*, and *θ*—and the elastic properties of the whole structure along the *x* or *y* axes can be determined using Equations (15) and (16) by considering the symmetrical properties.

## 3. Static Performance

**Figure 6:**
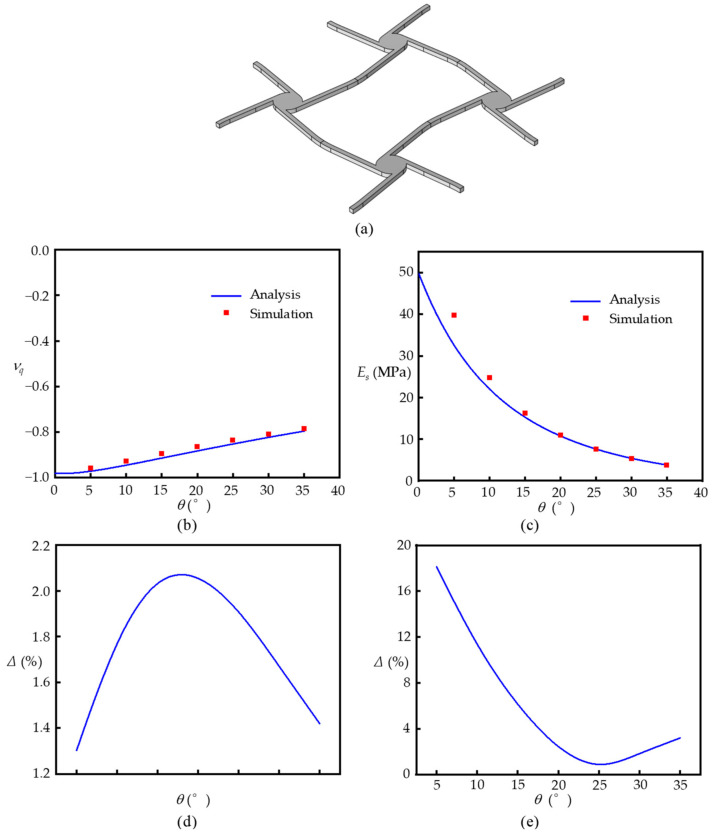
(**a**) Finite element model of unit cell. The analytical and numerical results of equivalent (**b**) Poisson’s ratio and (**c**) elastic modulus. Relative errors between analytical and numerical results of equivalent (**d**) Poisson’s ratios and (**e**) elastic modulus.

In order to verify the effectivity and accuracy of the equivalent elastic properties calculated using Equations (15) and (16), the finite element method is introduced to provide numerical simulation results for comparison. If the unit cell in Figure 1c is made of aluminum, its finite element model is shown in Figure 6a with the material properties and geometric parameters listed in Table 1. The unit cell produces 1% strain in the simulation. For the unit cell subjected to extension loading along the *y* direction, as shown in Figure 1c, its deformation can be numerically calculated by using the commercial software COMSOL Multiphysics to obtain the equivalent elastic properties. The finite element model in Figure 6a is meshed by the second-order tetrahedral element with size 1.2 × 10^−2^ mm. Among the geometric parameters in Equations (15) and (16), the tilt angle of ligament *θ* is more flexible to alter the equivalent elastic properties. Therefore, to change the tilt angle of the ligament *θ* from 5° to 35°, the varied equivalent Poisson’s ratios and elastic moduli are shown in Figure 6b,c, respectively.

For the equivalent Poisson’s ratios in Figure 6b, the analytical results (blue line) obtained using Equation (15) match very well with the numerical results (red square). The relative errors between them are shown in Figure 6d, which obtain a maximum of about 2% at a tilt angle θ=18°, demonstrating the high accuracy of Equation (15). For the equivalent elastic modulus in Figure 6c and relative errors in Figure 6e, the analytical results from Equation (16) are less than the analytical results at small tilt angles, but still obtain a high accuracy (relative errors of less than 5%) when the tilt angles are larger than θ=16°. Therefore, the proposed analytical equations can be used to theoretically predict the static mechanical properties of cross tetrachiral honeycomb metamaterials. It is noteworthy that the proposed cross tetrachiral honeycomb metamaterial has a good performance of negative Poisson’s ratio, which can be adjusted by altering the tilt angle *θ*.

In Figure 6b, the absolute value of equivalent negative Poisson’s ratio decreases as the tilt angle *θ* increases. As shown in Figure 2a, the small tilt angle of ligament *θ* can introduce a smaller vertical deformation along the tensile direction caused by ligaments AA’ and CC’, and the deformation can rotate the cylinder to induce a bending moment acting on ligaments BB’ and DD’ for providing horizontal deformation. Therefore, the equivalent negative Poisson’s ratio depends on the rotation of the cylinder to turn the vertical and horizontal displacements of deformed ligaments. With the gradually increasing tilt angle of the ligament, the bending deformations of ligaments AA’ and CC’ increase to cause a larger vertical displacement of the unit cell; however, the influence of the cylinder rotation on the horizonal displacement caused by ligaments BB’ and DD’ is limited. Therefore, the equivalent negative Poisson’s ratio can be adjusted by selecting a proper tilt angle *θ*.

For the equivalent elastic modulus, as shown in Figure 6c, there is a better consistency between the analytical results from Equation (16) and the numerical results at larger tilt angles. As the tilt angle *θ* increases, the equivalent elastic modulus becomes lower. This is because for a small tilt angle *θ*, the vertical displacement is mostly contributed by the longitudinally tensile deformations of ligaments AA’ and CC’, for which their stiffness is much larger than their bending stiffness, so that the equivalent elastic modulus is relatively big. On the contrary, the larger tilt angle can induce the vertical displacement with the bending-dominated deformations of ligaments AA’ and CC’, resulting in a larger deformation producing a smaller equivalent elastic modulus.

From Figure 6d,e, it can be seen that there is a difference between the theoretical predictions and the simulation results for Poisson’s ratio and elastic modulus, especially for the elastic modulus at smaller tilt angles *θ*. When *θ* is small, the ligaments are mainly subjected to tensile or compressive deformations by external loads. This causes the deformation of the ligament to be more dominated by axial deformation than bending deformation. However, the theoretical prediction is based on beam assumption, and only focuses on the bending deformation, neglecting the axial deformation. This is the reason why an error is caused between the theory and the simulation results at small tilt angles *θ*, while the axial deformation of the ligament is important and non-negligible. In addition, it demonstrates that the tilt angle *θ* is a useful parameter to change the equivalent static properties of cross tetrachiral honeycomb metamaterials.

**Figure 7:**
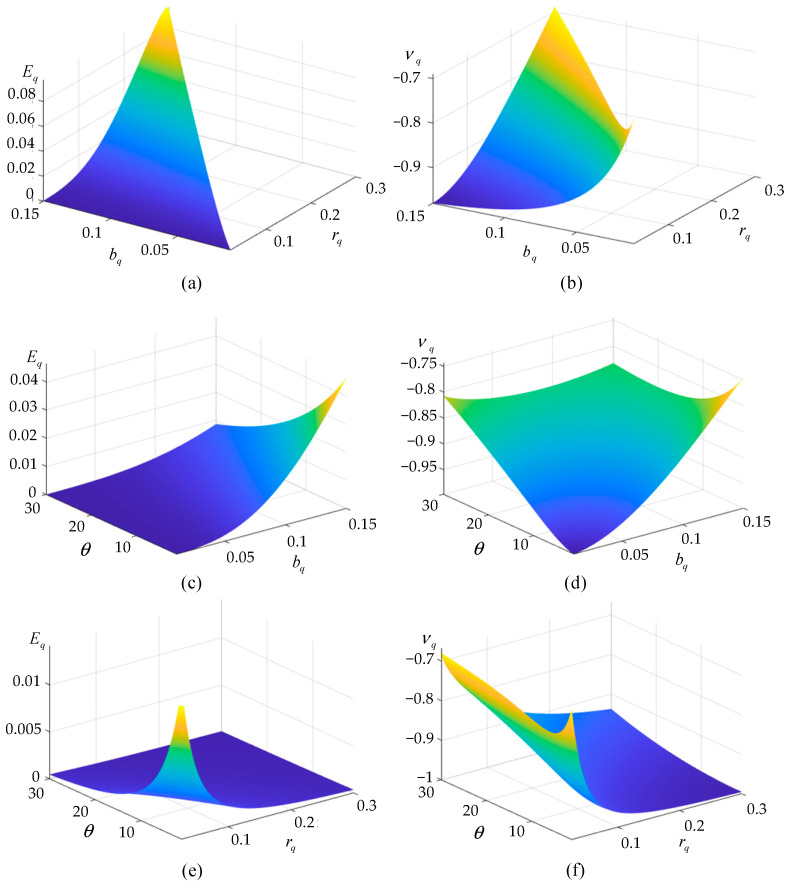
(**a**) Diagram of *E_q_* with respect to *b_q_* and *r_q_* when θ=8°. (**b**) Diagram of *υ_q_* with respect to *b_q_* and *r_q_* when θ=8°. (**c**) Diagram of *E_q_* with respect to *b_q_* and *θ* when *r_q_* = 0.1. (**d**) Diagram of *υ_q_* with respect to *b_q_* and *θ* when *r_q_* = 0.1. (**e**) Diagram of *E_q_* with respect to *r_q_* and *θ* when *b_q_* = 0.05. (**f**) Diagram of *υ_q_* with respect to *r_q_* and *θ* when *b_q_* = 0.05.

Since the analytical results are in good agreement with the numerical results, the equivalent elastic parameters *E_q_* and *ν_q_* of cross tetrachiral honeycomb metamaterials can be predicted using Equations (15) and (16). There are three independent dimensionless geometric parameters—*r_q_*, *b_q_*, and *θ*—which can be used to design the equivalent elastic parameters of the proposed metamaterial, as shown in Figure 7. If we set the tilt angle *θ* = 8°, *E_q_* and *ν_q_* can be altered by changing geometric parameters *r_q_* and *b_q_*, as shown in Figure 7a,b, respectively. Similarly, if we set *r_q_* = 0.1 or *b_q_* = 0.05 and change the other two parameters, *E_q_* and *ν_q_* will correspondingly vary, as shown in Figure 7c,d or Figure 7e,f. Therefore, the static mechanical properties of cross tetrachiral honeycomb metamaterials can be predicted by selecting suitable geometric parameters.

**Figure 8:**
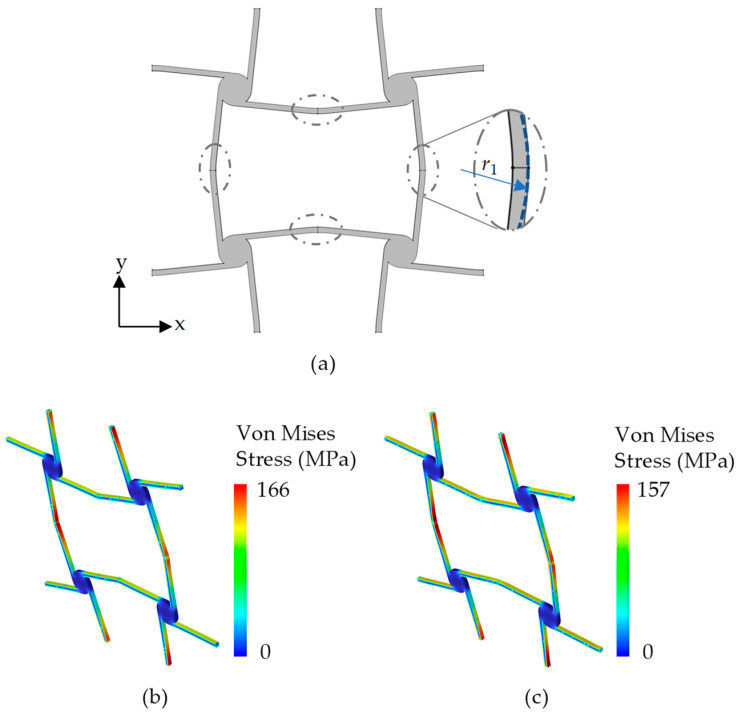
(**a**) Optimization of the folding angle of two adjacent ligaments. (**b**) Stress cloud image of the unit cell without optimization. (**c**) Stress cloud image of the unit cell with optimization.

Furthermore, it should be noticed that there is a stress concentration at the folding angle of two adjacent ligaments. In order to alleviate the stress concentration, the sharp folding angle is optimized into an arc curve with small curvature, as shown in Figure 8a. The stress distributions around the folding angle are calculated by using the commercial software COMSOL Mutiphysics 6.0, as shown in Figure 8b,c. The geometric parameters used in Figure 8a are the same as those in Table 1 and *r*_1_ = 20 mm. The unit cells in Figure 8b,c are subjected to a 1% tensile strain along the vertical direction. From Figure 8b,c, with the optimization, the maximum stress can be reduced from 166 MPa to 157 MPa with a reduction ratio of the maximum stress of about 5.42%, but the equivalent elastic modulus and Poisson’s ratio only change by about 1.05% and 0.24%, respectively. The results indicate that the small curvature at the folding angle of two adjacent ligaments does not make a big difference in the equivalent elastic parameters, but it can reduce the stress concentration at the folding angle to a certain extent. Actually, the folding angle curvature is closer to a straight line for meeting the requirements of machining in practical engineering, and can make the manufacturing process easier.

## 4. Dynamic Performance

As chiral metamaterials are highly applied in dynamic environments, it is very significant to investigate and adjust their dynamic properties. For the classical tetrachiral honeycomb metamaterial, as shown in Figure 1b, it is hard to design bandgaps in its band structure without any additional mass. Even if the metamaterial is made to produce a locally resonant bandgap by adding additional mass, the bandwidth will be narrow, and it will also be difficult to conveniently tune the bandgap. Therefore, how to manipulate the dynamic performance of cross tetrachiral honeycomb metamaterials is important to explore its potential in engineering applications.

### 4.1. Band Structures

For a linear elastic solid, its governing equation of wave motion with neglected body forces is as follows:

(17) $$\left(\lambda +\mu \right)\nabla \nabla \cdot u+\mu \nabla^{2}u=\rho \ddot{u},$$

where ρ is the mass density, λ and μ are the Lame constants, and **u** is the displacement vector, respectively. Considering a harmonic wave with angular frequency *ω* propagating in a two-dimensional O*xy* plane, the solution of Equation (17) can be written as follows:

(18) $$u=u_{k}e^{i\left(k\cdot r-\omega t\right)},$$

where $r=\left(x,y\right)$, $k=\left(k_{x},k_{y}\right)$ is the wave vector, and $u_{k}$ is the Bloch displacement vector.

By substituting Equation (18) into Equation (17), the wave propagation equation in the periodic structure can be rewritten as follows:

(19) $$\left[K\left(\lambda ,\mu ,k\right)-\omega M\left(\rho \right)\right]u_{k}=0.$$

To obtain a non-trivial solution of $u_{k}$ from Equation (19), an eigenvalue problem should be solved, i.e.,

(20) $$\left|K\left(\lambda ,\mu ,k\right)-\omega M\left(\rho \right)\right|=0$$

and the band structures of the periodic structure can be obtained.

**Figure 9:**
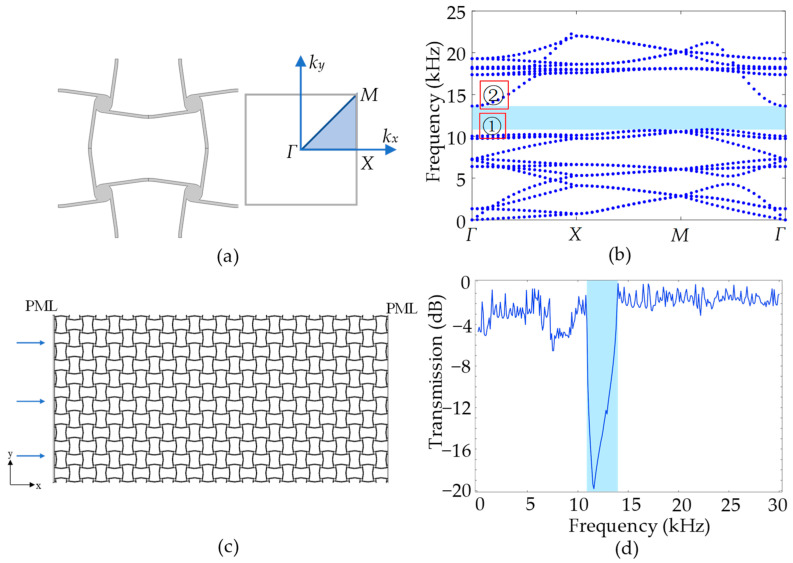
(**a**) The unit cell and the Brillouin zone. (**b**) The band structures. (**c**) The cross tetrachiral honeycomb structure. (**d**) The transmission ratio of the in-plane propagating wave in the structure.

For the same unit cell considered in a static case with the material properties and geometric parameters in Table 1, the dynamic performance of the cross tetrachiral honeycomb metamaterial is calculated using Equation (20) through the finite element commercial software COMSOL Multiphysics. To ensure the accuracy of the dynamic simulation, the finite element model is meshed by the second-order tetrahedral element with size 1.2 × 10^−2^ mm, which is less than one-tenth of the wavelength. Consider the in-plane case as the unit cell and its irreducible Brillouin zone shown in Figure 9a; if the tilt angle of ligament is selected as θ=8°, the band structures can be calculated and are shown in Figure 9b. For the dynamic case, even if the metamaterial undergoes an overall large deformation, the end part of the metamaterial retains a small deformation to satisfy the assumptions of the Euler–Bernoulli theory. In the simulation, during the unit cell with a 2% strain, the strain of each ligament is less than 0.56%.

As shown in Figure 9b, there is a wide bandgap in the band structures from a frequency of 10.8 kHz to 13.4 kHz, as marked in the blue shade. To verify the band structures, a two-dimensional cross tetrachiral honeycomb structure with 13×6 unit cells, as shown in Figure 9c, is introduced to calculate the transmission ratio. A longitudinal plane wave is excited along the left side with the displacement amplitude $U_{i}$, and the transmission signal along the right side with displacement amplitude $U_{i}$ is calculated using the finite element commercial software COMSOL Multiphysics. Perfect-matched layers (PMLs) are attached to both sides of the structure to eliminate the influence of reflected waves. The transmission ratio of the wave energy is calculated by $U_{t}^{2}/U_{i}^{2}$, and is shown in Figure 9d. The blue shade in Figure 9d marks the frequency range of the bandgap in Figure 9b, where a sharp drop in transmission ratios is observed. At other frequencies, the transmitted waves can propagate with a higher transmission ratio, which is consistent with the band structures.

**Figure 10:**
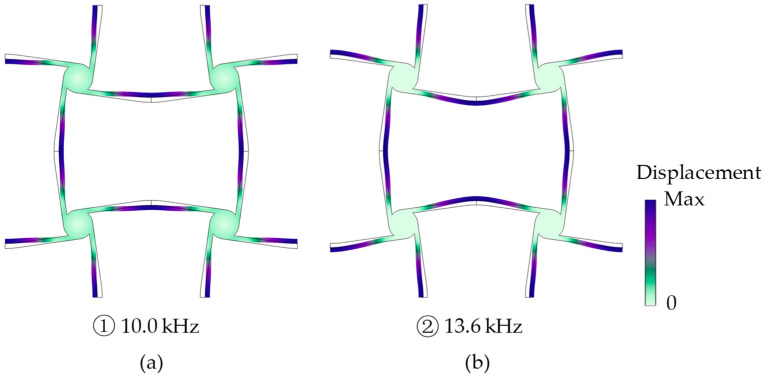
The mode shapes of unit cell at the (**a**) down and (**b**) up boundaries of the bandgap.

In order to analyze the generation of the bandgap, the wave modes at the up and down boundaries of the bandgap with a frequency of 10.0 kHz and 13.6 kHz (marked 1 and 2 at *Γ* in Figure 9b) are calculated using COMSOL, and the displacement amplitude of the unit cell is amplified and shown in Figure 10. In Figure 10, it can be noticed that the cylinders rotate rigidly and the ligaments move like cantilever beams with in-plane flexural deformations. Therefore, our simplified model in Section 2 can also be used in analyzing the dynamic case. The longitudinally incident wave propagates through the cross tetrachiral honeycomb structure to induce the flexural deformations of horizontal ligaments, which rotate cylinders to drive the flexural deformations of vertical ligaments; then, the wave moment transfers from one to another.

Comparing the mode shapes in Figure 10a,b, there is no big difference to be found in the vertical ligaments, but there is a phase difference π between the horizontal ligaments. The lowest bandgap is formed by a combination of locally resonant bandgaps, as shown in Figure 10. In Figure 10a, if we focus on one elementary unit, the flexural deformations of the horizontal and vertical ligaments are all following the rotation direction of the cylinder to present an easier excited wave mode at a lower frequency (10.0 kHz). However, for the elementary unit in Figure 10b, an opposite flexural deformation between the horizontal and vertical ligaments can be observed, which is a more difficult mode to be excited and has a higher frequency (13.6 kHz). As a result, a bandgap is generated between both wave modes.

### 4.2. Bandgap Influenced by Geometric Parameters

**Figure 11:**
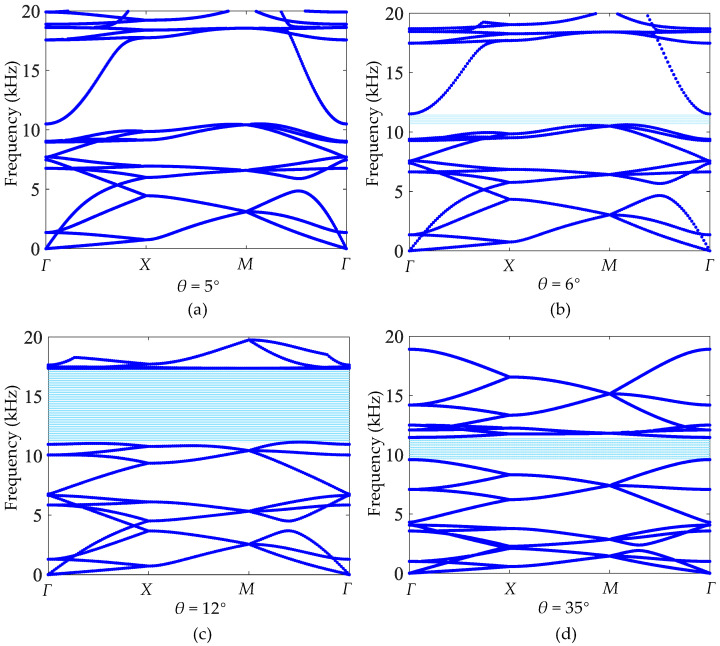
Band structures of different tilt angles: (**a**) θ=5°, (**b**) θ=6°, (**c**) θ=12°, (**d**) θ=35°.

To further explore the possibility of designing a cross tetrachiral honeycomb structure with specific dynamic properties, the influences of the geometric parameters of the unit cell are investigated one by one. Firstly, when the tilt angle of ligament *θ* is designed as *θ* = 5°, 6°, 12°, and 35°, the band structures are calculated, respectively, as shown in Figure 11a–d.

From Figure 11, it can be seen that there is no bandgap at θ=5°. The bandgap generates at θ=6°. Then, the bandwidth increases with the increasing *θ* until reaching 6.3 kHz (from 11.1 kHz to 17.4 kHz) at θ=12°. After that, the bandwidth will decrease. Based on the band structures in Figure 11, the frequency of the mode shape at the upper boundary is the main reason for the change in bandgap, which indicates that the mode shape of Figure 10b is greatly affected by the tilt angle of the ligament. In contrast, the frequency of the lower boundary is relatively stable, which varies slightly with increasing *θ*. Therefore, the bandgap of the cross tetrachiral honeycomb structure can be adjusted by changing the tilt angle of the ligament.

**Figure 12:**
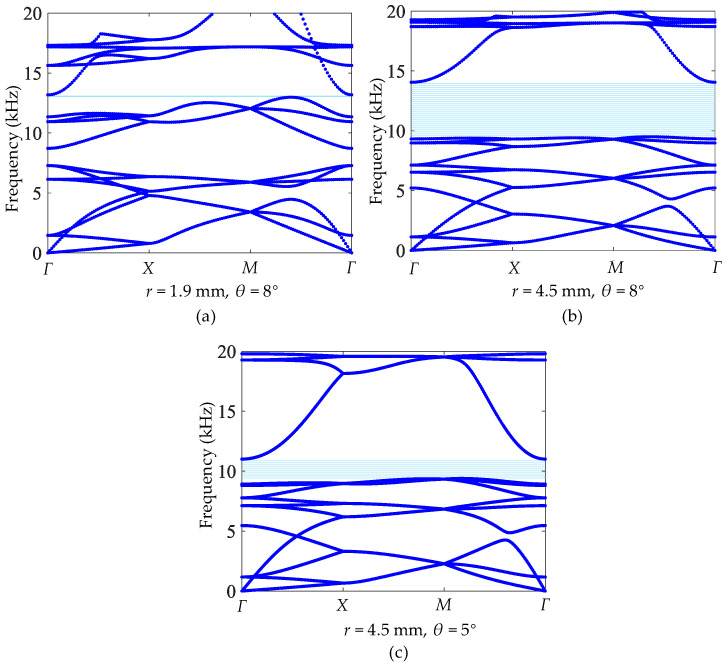
Band structures of different cylinder radii and tilt angles: (**a**) *r* = 1.9 mm, θ=8°. (**b**) *r* = 4.5 mm, θ=8°. (**c**) *r* = 4.5 mm, θ=5°.

Although the bandgap of the cross tetrachiral honeycomb structure is not generated by the local resonance of the cylinder, the change in cylinder radius can also vary the equivalent elastic modulus, as shown in Figure 7e; therefore, it also has an influence on the dynamic performance. For the same unit cell shown in Figure 6a, if we fix the tilt angle of the ligament to θ=8° and change the radius *r* of the cylinder from 1.9 mm to 4.5 mm, the band structures can be calculated and they are separately shown in Figure 12a,b. Comparing to the case shown in Figure 9a for *r* = 3 mm with bandwidth 2.6 kHz, the smaller radius *r* = 1.9 mm can induce a narrower bandgap with bandwidth 0.2 kHz, as shown in Figure 12a, and the larger one *r* = 4.5 mm can provide a wider bandgap with bandwidth 4.6 kHz, as shown in Figure 12b. In addition, as shown in Figure 7e, the equivalent elastic modulus will decrease when the radius *r* of the cylinder becomes larger, so the lower frequency of the bandgap will accordingly become lower from 13.0 kHz (*r* = 1.9 mm) to 9.5 kHz (*r* = 4.5 mm). However, the upper frequency of the bandgap is more stable, growing from 13.0 kHz to 14.1 kHz; therefore, the bigger radius *r* of the cylinder provides a wider bandgap. Nevertheless, for the radius *r* = 4.5 mm, if the tilt angle of the ligament *θ* is changed from 8° to 5°, the band structures, as shown in Figure 12c, demonstrate that the bandwidth of the bandgap is influenced by the tilt angle of the ligament *θ* and the radius *r* together.

**Figure 13:**
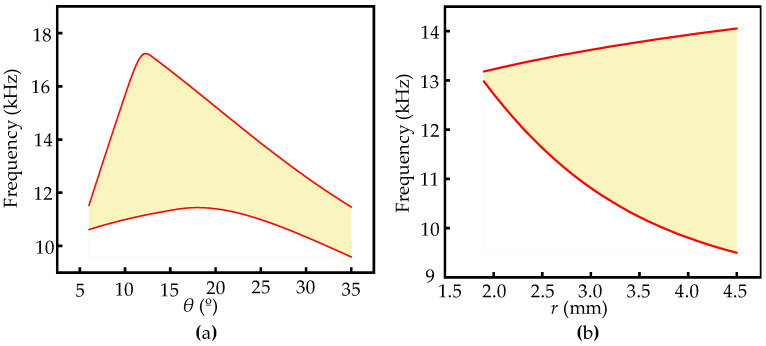
Bandgaps change with (**a**) the tilt angle of ligament *θ* and (**b**) radius *r*.

Since the tilt angle of the ligament *θ* and the radius of the cylinder *r* both have great influence on modulating the band structures, the relationships between the bandgap and selected geometric parameters should be investigated thoroughly. If the radius is fixed as *r* = 3 mm, the bandgap changed with the tilt angle of the ligament *θ*, as shown in Figure 13a. The bandgap does not exist at small tilt angles (θ<8°). It should be noted that the relationship between bandwidth and tilt angle are not monotonic and there is a maximum bandwidth (6.3 kHz) at a certain angle (θ=12°). The change in bandwidth is strongly dependent on the upper frequency of the bandgap with a rapid increase and gradual decrease, while the lower frequency of the bandgap alters smoothly. If the tilt angle of the ligament is fixed as θ=8°, the bandgap change with radius *r* is shown in Figure 13b. With the increase in cylinder radius *r*, the bandwidth also continuously increases. When *r* = 1.9 mm, the bandwidth is 0.2 kHz, and when the radius *r* reaches 4.5 mm, the bandwidth increases to 4.6 kHz. Therefore, the cross tetrachiral honeycomb metamaterial is very sensitive to the changes in geometric parameters, and its bandgap can be adjusted by the proper selection of these parameters.

### 4.3. Bandgap Changed by External Load

In addition to the adjustment of the geometric parameters, the external load can induce a large deformation of the cross tetrachiral honeycomb structure, and can also cause a change in the dynamic performance. This characteristic will be more significant in engineering because it does not need to change the structural configuration and can meet a variety of engineering requirements in dynamic behaviors of cross tetrachiral honeycomb structures.

**Figure 14:**
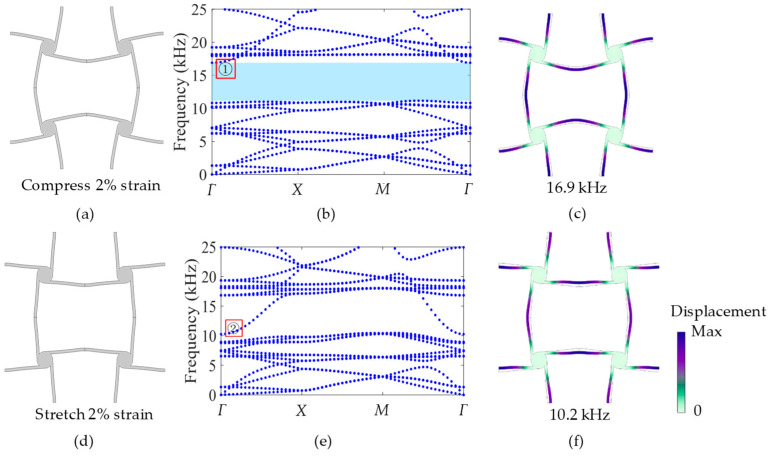
(**a**) Unit cell compressed by 2% strain. (**b**) Band structure after compression. (**c**) Mode shape of marked 1 at *Γ*, frequency 16.9 kHz. (**d**) Unit cell stretched by 2% strain. (**e**) Band structure after tension. (**f**) Mode shape of marked 2 at *Γ*, frequency 10.2 kHz.

For the same model shown in Figure 9a, when vertical compression acts on the unit cell to induce a −2% strain, as shown in Figure 14a, its band structure is calculated and shown in Figure 14b. Comparing with the band structure in Figure 9b without an external load, the bandgap can be changed from 10.8~13.4 kHz to 11.1~16.9 kHz, and the bandwidth correspondingly varies from 2.6 kHz to 5.8 kHz, which is 3.2 kHz wider than that without external load. This is because the compressive deformation can increase the tilt angle of the vertical ligament of the unit cell. Accordingly, the mode shape at the upper frequency of the bandgap, as marked 1 in Figure 14b, is calculated and is shown in Figure 14c; it has the same mode shape as Figure 10b. This means that no matter if the external compression exists or not, the mechanism of producing the bandgap is the same, but the frequency and bandwidth of the bandgap can be changed.

If the vertical tension is acting on the unit cell and induces a 2% strain, as shown in Figure 14d, its band structure is calculated and is shown in Figure 14e. It is clear in Figure 14e that there is no bandgap, because the tensive deformation can make the tilt angle of the vertical ligament become smaller. For comparison with the bandgap in Figure 9b, the mode shape marked as 2 in Figure 14e is calculated and shown in Figure 14f. It can be noticed that the mode shape in Figure 14f is the same as Figure 10a, which is the mode shape of the lower frequency of the bandgap without external tension. This demonstrates that the mechanism of bandgap modulation through deformation is the same as that through changing the tilt angle of the ligament.

**Figure 15:**
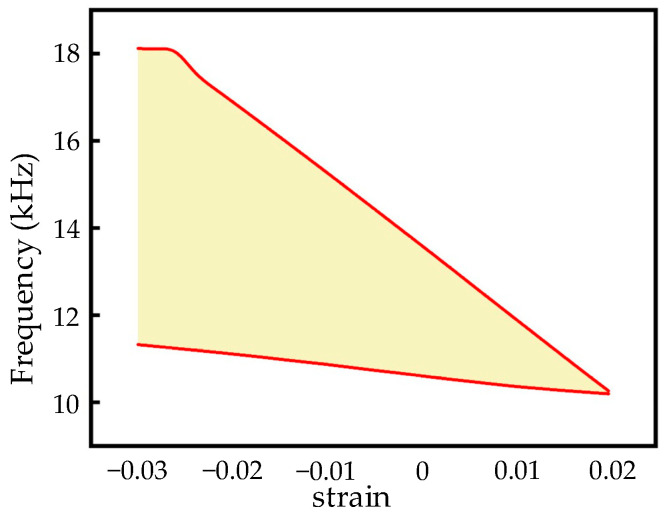
Bandwidth of bandgap varying with strain.

From Figure 14, it can be seen that the external load can effectively change the band structure and bandwidth of the bandgap, and can even convert the bandgap into the passband. In order to investigate the change in bandwidth with external load, the relationship between strain and bandgap is studied and is shown in Figure 15. In Figure 15, the results illustrate that with the increase in tensive strain, the bandgap gradually decreases and becomes narrower until it becomes passband at 2% strain; however, when the compressive strain works, the bandgap increases and becomes wider in order to reach the widest bandwidth at nearly 7 kHz. This confirms that the dynamic performance of the cross tetrachiral honeycomb structure has a very high sensitivity to external load.

In combination with the geometry of the unit cell after compression or tension in Figure 14a or Figure 14d, the reasons for the change in bandgap with strain can be analyzed. When the structure is vertically compressed, as in Figure 14a, the adjacent cylinders rotate harder in relative directions due to the external load, which makes the ligament between the cylinders more curved to form an arch. As a result, more energy is needed to stimulate the ligaments to produce the conversion of mode shape in Figure 14c, so the bandgap becomes wider at this time. When the structure is vertically stretched, the external load causes the adjacent cylinders to rotate oppositely, so that the ligaments between the cylinders become straight and gradually tend towards a straight line. As a result, less energy is required to stimulate the ligaments to keep the same mode shape as in Figure 14f, so the bandgap becomes narrower until it becomes a passband, which is a peculiar property of this structure.

## 5. Conclusions

A novel cross tetrachiral honeycomb metamaterial is proposed in this paper, and the combination of the cross chiral honeycomb structure and the tetrachiral honeycomb structure can be easily designed to reach the desired static and dynamic properties, particularly to achieve both excellent functions of negative Poisson’s ratio and wave isolation. Compared to the existing chiral honeycomb structures, the cross tetrachiral honeycomb metamaterial can break through the limitations of narrower and higher frequency bandgaps by introducing more tunable geometric parameters, and the integral design without additional masses can make the manufacturing process easy and precise.

Through theoretical analysis, the equivalent elastic modulus and Poisson’s ratio can be accurately predicted. In addition, these equivalent elastic parameters can be determined and adjusted by three geometric dimensionless parameters, which offer a significant convenience for the designable static performance of cross tetrachiral honeycomb structures in potential engineering applications.

The investigation of the dynamic properties of cross tetrachiral honeycomb metamaterials provides a tunable way to alter the bandgap for wave isolation. The influence of geometric parameters on the bandgap is studied, and the results indicate that the selection of the tilt angle of the ligament and the cylinder radius are important for adjusting the bandgap. Additionally, the external loading can also effect the change in bandgap from nonexistence to a wider one. With a small strain, the width of the bandgap can be changed greatly and linearly, and the bandgap can even be transformed into a passband. Therefore, the dynamic performance of cross tetrachiral honeycomb metamaterials is also designable.

In fact, the proposed metamaterial attempts to answer the research question about how to simultaneously satisfy both requirements of negative Poisson’s ratio and wide-frequency bandgap through a simple and low-cost design. Under some complex working conditions in engineering, the negative Poisson’s ratio and vibration isolation are required simultaneously.

In summary, the cross tetrachiral honeycomb metamaterial not only achieves a negative Poisson’s ratio effect, but can also result in excellent functions of vibration reduction and wave isolation. Therefore, the cross tetrachiral honeycomb metamaterial has designable static and dynamic performances by changing the geometric parameters or adjusting small linear deformations, and has broad prospects for engineering applications.

## Figures and Tables

**Table 1:** Material properties and geometric parameters.

*E* (GPa)	*υ*	*ρ* (kg/m^3^)	*t* (m)	*b* (m)	*l* (m)	*r* (m)	*A* (m^2^)	*I* (m^4^)
70	0.33	2700	0.001	0.001	0.06	0.003	10^−6^	$8.33\times 10^{-14}$

## Data Availability

The data presented in this study are available on request from the corresponding author.
